# Improving performance of 3D speckle tracking in arterial hypertension and paroxysmal atrial fibrillation by using novel strain parameters

**DOI:** 10.1038/s41598-019-43855-7

**Published:** 2019-05-14

**Authors:** G. Esposito, P. Piras, A. Evangelista, V. Nuzzi, P. Nardinocchi, G. Pannarale, C. Torromeo, P. E. Puddu

**Affiliations:** 1 Dipartimento di Scienze Cardiovascolari, Respiratorie, Nefrologiche, Anestesiologiche e Geriatriche, Sapienza –Università di Roma, Rome, Italy; 2 Dipartimento di Ingegneria Strutturale e Geotecnica, Sapienza – Università di Roma, Rome, Italy; 3 Ospedale San Giovanni Calibita Fatebenefratelli – Isola Tiberina, Rome, Italy

**Keywords:** Cardiac hypertrophy, Atrial fibrillation

## Abstract

The function of left atrium (LA) is closely related to LA remodeling and one of the most important mechanisms is an increased deposition of fibrous tissue that often is the basis for LA electro-mechanical changes before the onset of atrial fibrillation (AF). This study evaluated LA shape and function, by investigating standard and novel strain parameters calculated by a new approach based on homologous times derived from 3D speckle tracking echocardiography (3DSTE) in hypertensive (HT) and paroxysmal atrial fibrillation (PAF) patients with or without left ventricular hypertrophy (LVH), compared to control (C) subjects. LA function was assessed using homologous times to compare strain variables among different individuals, acquired at different physiological time periods. Standard global longitudinal (GLS) and circumferential (GCS) strains were measured at peak of atrial diastole, while longitudinal and circumferential strains (GLSh, GCSh), strain rate (GLSr, GCSr), volume (Vh) and volume rate (Vr) were measured during the atrial telediastolic phase (fifth homologous time) and atrial pre-active phase (tenth homologous time). Using ANOVA, we found an impaired LA deformation detected by standard, interpolated strains and strain rates in both HT and PAF groups compared to C. We also performed ROC analysis to identify different performances of each parameter to discriminate groups (GLSr10 + GCSr10: C vs PAF 0.935; C vs PAF_LVH 0.924; C vs HT_LVH 0.844; C vs HT 0.756). Our study showed anatomical and functional LA remodeling in patients with PAF and HT. 3D strains and strain rates derived from the homologous times approach provide more functional information with improved performance to identify among the explored groups, in particular PAF patients.

## Introduction

Atrial fibrillation (AF) is the most common arrhythmia in the general population. The prevalence of AF is 1–2%^1–3^ and its complications have a very high socio-economic impact^4,5^ due to a close relationship with ischemic stroke, embolisms, heart failure, hospitalizations and death risk along with worse quality of life, lower exercise tolerance and impaired left ventricular (LV) function^6–8^. Paroxysmal atrial fibrillation (PAF) is mainly asymptomatic^8^ and diagnosis is based on long-term electrocardiographic (ECG) monitoring. However, routine use of long-term ECG monitoring is rarely possible for economic and logistic reasons, so that PAF diagnosis is still challenging before symptoms’ onset^4–9^. However, prompt identification of patients with previous silent AF may be strategic for preventing purposes.

As it is reasonable that atrial and ventricular functions are related^10–12^, it is also much possible that conditions such as hypertension (HT) and AF are also related and it is easy to observe that HT is concomitant with or precedes the development of PAF. Both PAF and HT have a common thread, consisting of strongly modified electrical and mechanical characteristics^13,14^ which may both follow an altered extracellular matrix composition as the result of increased fibrotic tissue deposition detectable by magnetic resonance^15,16^. Therefore, atrial structure and function evaluation by imaging, is a useful tool in order to identify patients with impaired mechanical properties and increased stiffness or reduced LA diastolic relaxation. In recent years many parameters have emerged as “vertical asymmetry” and “LA sphericity” for a better evaluation of atrial remodeling as a predictor of AF recurrence after ablation^17,18^, but none of these evaluated LA function.

Concomitantly with impaired mechanical function, LA undergoes an anatomical remodeling with larger volume and reduced function^19,20^, a process that may either start or maintain AF^16^. A novel approach with three-dimensional speckle tracking echocardiography (3DSTE) offers the advantage to assess LA structure better than 2D echocardiography^20^ when compared with magnetic resonance^21–24^ in which late-gadolinium MRI provides tissue information, but the relation between MRI intensity hyper-enhancement and underlying tissue pathophysiology (including relation with different types of fibrosis, interstitial edema, etc.) is far from being well understood. Anatomical remodeling is better evaluated using shape parameters like volume or diameter measured by standard 2D echocardiography, whereas function is more appropriately investigated by extents of deformation assessed by 3DSTE^25–28^. The left atrial volume index (LAVi) calculated in 2D is one of the most used shape parameters for the ability to predict the likelihood of new AF onset or recurrence after cardioversion/ablation, in PAF patients^4,17,18^, or to establish the severity of cardiac remodeling in patients with HT. It is usually evaluated at atrial diastole and it has a prognostic value with a high clinical impact.

In the present investigation, we tested the performance of one of the possible LA multi-modal image studies to identify patients in sinus rhythm, with HT and an asymptomatic episode of PAF, by comparing them with strains, strain’s rates, 3D LA end-diastolic volume (LAEDV3D), 3D LA end-systolic volume (LAESV3D) and LAVi. Using the homologous times’ concept^29–31^ we implemented standard 3DSTE parameters obtaining global longitudinal and global circumferential strains (GLSh, GCSh) and their corresponding rates (Vr, GLSr, GCSr). Among the entire LA cycle we focused on atrial diastole (fifth homologous time) and pre-active time (tenth homologous time); at these times we investigated the relationship among HT and PAF with or without left ventricular hypertrophy (LVH) in terms of LA function and shape.

## Results

### Clinical characteristics

Demographic, clinical and 2D echocardiographic characteristics of the population under study are reported in Tables 1, 2. We included 130 individuals in the study. Groups were composed of 82 normal subjects (Control), 9 patients with arterial hypertension (HT) and paroxysmal episodes of atrial fibrillation (AF), without left ventricular hypertrophy (LVH) (PAF group), 11 patients with PAF, HT and LVH (PAF_LVH group), 10 patients with HT and LVH (HT_LVH group) and 18 patients with HT without LVH (HT group). There were significant age differences amongst the 5 groups (age ± SD): Controls were the youngest while in the PAF_LVH group there were the oldest patients. In PAF group, CHA2DS2-VASc score was between 0–1 in about 66% and 2–3 in nearly 33%. However, in PAF_LVH nearly 81% had a CHA2DS2-VASc score between 2–3. For the purpose of this analysis no subject had diabetes of any type; 57% and 44% of PAF patients received anticoagulation treatment and antiarrhythmic drugs. In PAF_LVH group, these proportions were higher than in the other groups (91% and 73% respectively). Nearly 74% of the patients with HT received ACE-I or ARB, and >50% a beta-blocker.

**Table 1:** Demographic attributes, cardiovascular risk factors and pharmacological therapy of the study population subdivided into groups.

Clinical Features	Controls N = 82		PAF N = 9		HT N = 18		HT_LVH N = 10		PAF_LVH N = 11		P-value
	Mean ± sd	n	Mean ± sd	N	Mean ± sd	N	Mean ± sd	n	Mean ± sd	n	
Gender (M/F)	48 (59)/34 (42)	n = 82	8 (89)/1 (11)	n = 9	10 (56)/8 (44)	n = 18	9 (90)/1 (10)	n = 10	6 (55)/5 (46)	n = 11	0.131
Age (years)	54.30 ± 11.17	n = 81	59.78 ± 13.8	n = 9	56.39 ± 7.58	n = 18	57.5 ± 9.62	n = 10	71.64 ± 3.96	n = 11	<0.001*
Weight (Kg)	71.44 ± 12.26	n = 66	84.89 ± 10.2	n = 9	75.94 ± 9.72	n = 18	82.1 ± 11.02	n = 10	74.45 ± 13.3	n = 11	0.004*
Height (m)	1.73 ± 0.1	n = 66	1.76 ± 0.1	n = 9	1.71 ± 0.09	n = 18	1.73 ± 0.07	n = 10	1.7 ± 0.12	n = 11	0.623
BMI (Kg/m2)	23.8 ± 3.12	n = 66	27.42 ± 2.2	n = 9	26.09 ± 2.94	n = 18	27.37 ± 2.61	n = 10	25.76 ± 4.11	n = 11	<0.001*
SBP (mmHg)	116.41 ± 9.61	n = 64	127.78 ± 11.76	n = 9	129.72 ± 8.31	n = 18	129.5 ± 10.66	n = 10	128.18 ± 10.79	n = 11	<0.001*
DBP (mmHg)	73.98 ± 7.72	n = 64	79.44 ± 3.91	n = 9	82.78 ± 7.12	n = 18	81.5 ± 7.47	n = 10	73.64 ± 5.05	n = 11	<0.001*
BP Average (mmHg)	68.78 ± 37.33	n = 82	95.55 ± 5.71	n = 9	98.43 ± 6.8	n = 18	97.5 ± 8.21	n = 10	91.82 ± 5.65	n = 11	<0.001*
BSA (m²)	1.84 ± 0.19	n = 66	2.02 ± 0.19	n = 9	1.87 ± 0.15	n = 18	1.96 ± 0.15	n = 10	1.84 ± 0.21	n = 11	0.046*
CHA2DS2-VASc (0–1) (%)	80 (98)	n = 82	0 (0)	n = 9	8 (44)	n = 18	6 (60)	n = 10	2 (18)	n = 11	0.002*
CHA2DS2-VASc (2–3) (%)	2 (2)	n = 82	9 (100)	n = 9	9 (50)	n = 18	4 (40)	n = 10	9 (81)	n = 11	0.106
CHA2DS2-VASc (4) (%)	0 (0)	n = 82	0 (0)	n = 9	1 (6)	n = 18	0 (0)	n = 10	0 (0)	n = 11	**NA**
Hypertension (%)	0 (100)	n = 82	9 (100)	n = 9	18 (100)	n = 18	10 (100)	n = 10	11 (100)	n = 11	<0.001*
Dyslipidemia (%)	0 (100)	n = 82	1 (11)	n = 9	4 (22)	n = 18	4 (40)	n = 10	0 (100)	n = 11	<0.001*
Smoke (%)	16 (20)	n = 82	0 (100)	n = 9	5 (28)	n = 18	2 (20)	n = 10	0 (100)	n = 11	0.416
Familiarity (%)	9 (11)	n = 82	0 (100)	n = 9	10 (56)	n = 18	3 (30)	n = 10	2 (18)	n = 11	<0.001*
Diabete (%)	0 (100)	n = 82	0 (100)	n = 9	0 (100)	n = 18	0 (100)	n = 10	0 (100)	n = 11	1
**Medications**											
Ace-Is (%)	0 (100)	n = 82	3 (33)	n = 9	3 (17)	n = 18	4 (40)	n = 10	5 (46)	n = 11	<0.001*
Nitr (%)	0 (100)	n = 82	0 (100)	n = 9	0 (100)	n = 18	0 (100)	n = 10	0 (100)	n = 11	1
Beta Blockers (%)	0 (100)	n = 82	6 (67)	n = 9	7 (39)	n = 18	6 (60)	n = 10	4 (36)	n = 11	<0.001*
Diuretics (%)	0 (100)	n = 82	2 (22)	n = 9	7 (39)	n = 18	2 (20)	n = 10	3 (27)	n = 11	<0.001*
Statine (%)	0 (100)	n = 82	1 (11)	n = 9	4 (22)	n = 18	5 (50)	n = 10	3 (27)	n = 11	<0.001*
Anticoagulation (%)	0 (100)	n = 82	5 (57)	n = 9	0 (100)	n = 18	0 (100)	n = 10	10 (91)	n = 11	<0.001*
Anti-Arrhythmics (%)	0 (100)	n = 82	4 (44)	n = 9	0 (100)	n = 18	0 (100)	n = 10	8 (73)	n = 11	<0.001*
Aspirin (%)	0 (100)	n = 82	0 (100)	n = 9	2 (11)	n = 18	3 (30)	n = 10	0 (100)	n = 11	<0.001*
ARB (%)	0 (100)	n = 82	3 (33)	n = 9	10 (57)	n = 18	3 (30)	n = 10	4 (36)	n = 11	<0.001*
CCB (Dihydropyridine) (%)	0 (100)	n = 82	0 (100)	n = 9	6 (33)	n = 18	2 (20)	n = 10	0 (100)	n = 11	<0.001*
Alfa Blockers -(%)	0 (100)	n = 82	0 (100)	n = 9	0 (100)	n = 18	1 (10)	n = 10	0 (100)	n = 11	0.017*

SBP - Systolic Blood Pressure; DBP – Diastolic Blood Pressure; BMI - Body Mass Index; BSA - Body Surface Area; ACE-Is – Ace-Inhibitors; NITR– nitrate; ARB – Angiotensin Receptor Blockers. CCB – Calcium channel blockers. NA – not applicable. ^*^Significance of P-value was set at <0.05 and refers to Kruskall-Wallis non parametric test; N indicates the total number of individuals for each group; n indicates the actual cases number for each variable thus excluding casewise missing data.

**Table 2:** Per-group 2D Echocardiographic variables.

2D Echocardiography	Control N = 82		PAF N = 9		HT N = 18		HT_LVH N = 10		PAF_LVH N = 11		P-value
	Mean ± sd	n	Mean ± sd	n	Mean ± sd	n	Mean ± sd	n	Mean ± sd	n	
LVEDD (cm)	47.21 ± 4.84	n = 66	49.89 ± 4.37	n = 9	47.5 ± 5.18	n = 16	52.5 ± 5.68	n = 10	51.82 ± 3.57	n = 11	0.002*
LVESD (cm)	30.17 ± 5.67	n = 66	31.11 ± 4.99	n = 9	27.44 ± 6.31	n = 16	32.4 ± 8.78	n = 10	29.45 ± 4.87	n = 11	0.293
IVSd (cm)	8.2 ± 1.45	n = 66	9.44 ± 1.81	n = 9	9.41 ± 1.42	n = 17	11.6 ± 1.17	n = 10	11.64 ± 2.11	n = 11	<0.001*
LVMASSi (g/m²)	87.55 ± 27.99	n = 66	90.39 ± 36.36	n = 9	80.89 ± 32.34	n = 18	137.27 ± 23.09	n = 10	156.64 ± 28.15	n = 11	<0.001*
PTDp (mmHg)	3.5 ± 1.3	n = 26	2.67 ± 1.12	n = 9	2.59 ± 0.94	n = 17	2.8 ± 1.99	n = 10	3.55 ± 1.69	n = 11	0.151
E (cm/s)	80.24 ± 20.03	n = 66	64.67 ± 16.28	n = 9	75 ± 20.86	n = 18	74.4 ± 17.82	n = 10	66.73 ± 13.47	n = 11	0.068
A (cm/s)	60.35 ± 15.03	n = 66	71.33 ± 13.5	n = 9	74.17 ± 18.19	n = 18	80.2 ± 12.18	n = 10	76.64 ± 8.85	n = 11	0.001*
E/A	1.37 ± 0.350	n = 66	0.92 ± 0.328	n = 9	1.03 ± 0.34	n = 18	1.109 ± 0.35	n = 10	0.82 ± 0.20	n = 11	<0.001*
DECT (msec)	201.62 ± 44.84	n = 66	221 ± 51.54	n = 9	224.67 ± 55.65	n = 18	212.2 ± 69.24	n = 10	220.91 ± 59.16	n = 11	0.383
E1LAT (cm/s)	16 ± 3.55	n = 66	10.89 ± 2.26	n = 9	11.76 ± 3.15	n = 17	12.7 ± 2.83	n = 10	11.27 ± 3.1	n = 11	<0.001*
SLAT (cm/s)	11.88 ± 2.38	n = 66	12.44 ± 3.84	n = 9	11.12 ± 3.14	n = 17	11.9 ± 3.18	n = 10	11 ± 2.61	n = 11	0.646
E.E1LAT	5.26 ± 1.51	n = 66	6 ± 1.22	n = 9	6.88 ± 1.73	n = 17	6.4 ± 1.84	n = 10	6.45 ± 1.86	n = 11	0.001*
E/e’(m)	5.78 ± 1.44	n = 66	6.431 ± 2.31	n = 9	7.35 ± 1.76	n = 17	7.53 ± 2.41	n = 10	7.23 ± 1.81	n = 11	<0.001*
E1MED (cm/s)	12.42 ± 2.45	n = 66	9.56 ± 2.46	n = 9	9.33 ± 2.5	n = 18	8.2 ± 1.75	n = 10	8.27 ± 2.37	n = 11	<0.001*
SMED (cm/s)	9.8 ± 1.23	n = 66	9.38 ± 1.3	n = 8	9.44 ± 1.69	n = 18	8.8 ± 1.93	n = 10	9.45 ± 1.57	n = 11	0.287
SDX (cm/s)	14.41 ± 1.94	n = 66	14.11 ± 2.26	n = 9	14 ± 2.68	n = 18	12.7 ± 2.11	n = 10	13.7 ± 3.43	n = 10	0.255
LVEDVi (ml/m²)	56.83 ± 13.63	n = 66	59.39 ± 8.95	n = 9	57.15 ± 16.56	n = 18	66.96 ± 13.18	n = 10	65.65 ± 7.87	n = 11	0.088
LVESVi (ml/m²)	22.62 ± 5.41	n = 66	25.32 ± 7.27	n = 9	22.48 ± 7.08	n = 18	30.8 ± 7.84	n = 10	33.08 ± 14.22	n = 11	<0.001*
LVEF (%)	60.36 ± 5.12	n = 66	60.11 ± 4.46	n = 9	60.39 ± 5.08	n = 18	55.7 ± 3.86	n = 10	60 ± 5.1	n = 10	0.102
VDX_ADX (mmHg)	22.26 ± 6.37	n = 57	25.67 ± 4.8	n = 9	24.67 ± 7.55	n = 18	25.33 ± 4.72	n = 9	27 ± 8.6	n = 10	0.148
PASP (mmHg)	26.31 ± 7.14	n = 51	30.78 ± 5.26	n = 9	30.44 ± 8.16	n = 16	31.22 ± 4.74	n = 9	31.55 ± 7.66	n = 11	0.040*

LVEDD – left ventricle end diastolic diameter; LVESD – left ventricle end systolic diameter; IVSd – intraventricular septum; LVMASSi – left ventricle mass index; PTDp – pulmonary telediastolic pressure; DECT – deceleration time; LVEDVi – left ventricle end diastolic volume index; LVESVi – left ventricle end systolic volume index; LVEF – left ventricle ejection fraction; PASP – pulmonary artery systolic pressure; LAVi – left atrial volume index; VDX_ADX – right atrium ventricular gradient; ^*^Significance of P-value was set at <0.05 and refers to Kruskall-Wallis non parametric test; N indicates the total number of individuals for each group; n indicates the actual cases number for each variable thus excluding casewise missing data.

### Two dimensional echocardiographic characteristics of groups

Concerning 2D parameters, LV end-systolic diameter (LVESD), LV end-diastolic volume index (LVEDV) and ejection fraction (LVEF) were not statistically different among groups. Left ventricular mass index (LVMASSi) and interventricular septum (IVSd) were higher in the HT_LVH and PAF_LVH groups in comparison with C (p-value < 0.001). Systolic function assessment by tissue Doppler imaging did not show significant differences in S lateral wave, S septal wave and right ventricle S wave among groups. LV filling pressure indicated that E wave and deceleration time were similar in all patients (p-value = 0.383). A wave, E/e’ ratio, septal e’ wave and lateral e’ wave were significantly different in each group but within the normal range. Lastly, diastolic pulmonary pressure (PTDp) and AV peak gradient (VDX-ADX), were not significantly different. (see Table 2). No patient showed any wall motion abnormality or significant valvular disease.

### Left atrial study

LAVi, LAESV3D and LAEDV3D were higher in each group compared to Control. Specifically, they were different within the groups with progressive increasing values from Control to PAF_LVH (Control → HT → HT_LVH → PAF → PAF_LVH) (p-value < 0.001*) (see Table 1).

Default 3DSTE parameters were statistically significant among Control versus the other groups, (GLS: p-value = 0.004; GCS: p-value =<0.001), while the homologous times’ approach showed that global longitudinal strain (GLSh5), global circumferential strain (GCSh5), and volume (Vh5) were significantly different among all groups at atrial diastole (fifth homologous times), while only Volume (Vh10) was statistically significant at pre-active time (tenth homologous). (see Table 3). Concerning strain rate parameters, global longitudinal strain rate (GLSr10), global circumferential strain rate (GCSr10), and Volume rate (Vr10) were different, among categories, at pre active time, while only global circumferential strain rate (GCSr10) was significant at atrial diastole. Under Kruskal Wallis test LAVi differed between Control and the others groups (p-value < 0.001), while LAESV3D and LAEDV3D showed a different pattern of significance (see Supplementary Table 1) in pairwise comparisons. The same test was performed for standard 3DSTE (GCS and GLS) and differences were found among Control, PAF and PAF_LVH (see Supplementary Table 1). Kruskal Wallis test performed on novel derived parameters (strain, strain rate, volume and volume rate) at atrial diastole and at pre active time, showed different results among Control and the other groups. These findings underline the presence of a progressive impaired LA function caused by LA remodeling in HT and PAF patients; in particular, the greater was the remodeling, the more was the dysfunction (see Tables 3, 4).

**Table 3:** Kruskall Wallis test between left atrial volume index, standard 3D strain and 3D strain rate variables calculated by homologous times approach of population divided by groups.

	Control N = 82		PAF N = 9		HT N = 18		HT_LVH N = 10		PAF_LVH N = 11		P-value
	Mean ± sd	n	Mean ± sd	n	Mean ± sd	n	Mean ± sd	n	Mean ± sd	n	
**Echocardiographic Atrial Variables**											
**2D ECHOCARDIOGRAPHY**											
LAVi (ml/m²)	19.79 ± 6.46	66	29.36 ± 6.97	9	24.15 ± 11.45	18	28.17 ± 5.61	10	39.3 ± 10.74	11	<0.001*
**Standard 3D strain variables**											
GCS (%)	33.07 ± 13.51	82	20.31 ± 7.95	9	28.81 ± 10.22	18	26.26 ± 4.42	9	19.59 ± 11.02	10	<0.001*
GLS (%)	27.55 ± 8.02	82	23.69 ± 3.37	9	24.43 ± 5.05	18	24.43 ± 6.76	10	19.50 ± 5.12	11	0.004*
LAESV3D (ml)	21.05 ± 7.82	66	33.08 ± 07.69	9	27.96 ± 12.32	16	28.75 ± 09.48	10	40.88 ± 17.44	11	<0.001*
LAEDV3D (ml)	45.73 ± 14.69	66	60.42 ± 10.95	9	55.76 ± 15.90	17	56.92 ± 20.67	10	67.73 ± 21.59	11	<0.001*
**3D Strain Variables At Homologous Times**											
*Tenth Homologous Time (Pre Active Time)*											
GCSh10 (%)	18.029 ± 10.115	82	15.213 ± 8.913	9	21.624 ± 8.641	18	18.89 ± 4.793	10	15.76 ± 10.809	11	0.968
GLSh10 (%)	14.431 ± 6.309	82	15.836 ± 3.654	9	17.028 ± 3.456	18	16.223 ± 4.655	10	14.226 ± 4.463	11	0.351
Vh10 (ml)	33.542 ± 11.998	82	51.874 ± 8.283	9	47.104 ± 14.255	18	50.784 ± 15.744	10	61.432 ± 19.092	11	<0.001*
*Fifth Homologous Time (Atrial Diastole)*											
GCSh5 (%)	31.967 ± 13.313	82	17.261 ± 8.664	9	17.261 ± 8.664	9	24.502 ± 5.35	10	16.661 ± 11.174	11	<0.001*
GLSh5 (%)	26.432 ± 8.239	82	23.083 ± 3.361	9	23.075 ± 5.263	18	23.688 ± 6.698	10	18.933 ± 5.113	11	0.002*
Volume (Vh5) (ml)	44.85 ± 14.917	82	58.673 ± 11.602	9	53.906 ± 16.229	18	59.293 ± 16.783	10	65.127 ± 22.496	11	<0.001*
**3D Strain Rate Variables At Homologous Times**											
*Tenth Homologous Time (Pre Active Time)*											
GCSr10 (%/sec)	−0.163 ± 0.077	82	−0.05 ± 0.028	9	−0.069 ± 0.05	18	−0.06 ± 0.036	10	−0.048 ± 0.039	11	<0.001*
GLSr10 (%/sec)	−0.14 ± 0.052	82	−0.075 ± 0.018	9	−0.083 ± 0.038	18	−0.073 ± 0.037	10	−0.047 ± 0.033	11	<0.001*
VSr10 (ml/sec)	−13.17 ± 05.04	82	−8.84 ± 3.06	9	−9.12 ± 5.05	18	−9.11 ± 5.63	10	−6.37 ± 5.71	11	<0.001*
*Fifth Homologous Time (Atrial Diastole)*											
GCSr5 (%/sec)	0.063 ± 0.049	82	0.043 ± 0.04	9	0.038 ± 0.046	18	0.049 ± 0.027	10	0.043 ± 0.039	11	0.044*
GLSr5 (%/sec)	0.036 ± 0.044	82	0.029 ± 0.018	9	0.036 ± 0.031	18	0.037 ± 0.022	10	0.035 ± 0.02	11	0.953
Vr5 (ml/sec)	5.048 ± 3.628	82	5.357 ± 3.477	9	4.743 ± 4.095	18	6.267 ± 4.124	10	6.344 ± 4.169	11	0.269

LAVi – Left atrial volume index; GCS – Global circumferential strain; GLS – Global longitudinal strain; LAESV3D – Left atrial end systolic volume 3D; LAEDV3D – Left atrial end diastolic volume 3D; Vh10- Volume at tenth homologous time; Vr10- Volume rate at tenth homologous times; Vh5- Volume at fifth homologous time; Vr5- Volume rate at fifth homologous time; GCSh10- Global circumferential strain at tenth homologous times; GLSh10 – Global longitudinal strain at tenth homologous time; GCSh5 – Global circumferential strain at fifth homologous time; GLSh5 – Global longitudinal strain at fifth homologous time; GCSr10 – Global circumferential strain rate at tenth homologous time; GLSr10 – Global longitudinal strain rate at tenth homologous time; GCSr5 – Global circumferential strain rate at fifth homologous time; GLSr5 – Global longitudinal strain rate at fifth homologous time; N indicates the total number of individuals for each group; n indicates the actual cases number for each variable thus excluding casewise missing data; *Significance of P-value was set at <0.05.

**Table 4:** AUC of 3D strain parameters obtained by homologous times approach compared with LAVi (in the first row) and standard 3D strain to identifying each pathology.

Variables/Groups	Control/PAF	Control/HT	Control/HT_LVH	Control/PAF_LVH	PAF/HT	PAF/HT_LVH	PAF/PAF_LVH	HT/HT_LVH	HT/PAF_LVH	HT_LVH/PAF_LVH
LAVi	0.779	0.797	0.806	0.916	0.620	0.653	0.808	0.662	0.729	0.910
Vh10	0.816	0.666	0.709	0.796	0.648	0.655	0.722	0.617	0.689	0.644
Vr10	0.647	0.661	0.625	0.688	0.625	0.648	0.686	0.618	0.645	0.638
GCSh10	0.617	0.612	0.626	0.610	0.626	0.680	0.655	0.616	0.641	0.746
GLSh10	0.623	0.632	0.609	0.628	0.621	0.632	0.627	0.652	0.627	0.686
GCSh10 + GLSh10	0.628	0.610	0.613	0.634	0.621	0.647	0.641	0.616	0.631	0.665
GCSr10	0.901	0.745	0.821	0.833	0.655	0.727	0.645	0.623	0.635	0.636
GLSr10	0.845	0.744	0.769	0.895	0.676	0.782	0.765	0.626	0.668	0.753
GCSr10 + GLSr10	0.937	0.744	0.850	0.925	0.671	0.708	0.710	0.699	0.638	0.639
Vh5	0.673	0.613	0.628	0.720	0.646	0.704	0.648	0.626	0.699	0.654
Vr5	0.617	0.614	0.620	0.733	0.635	0.759	0.640	0.623	0.734	0.646
GCSh5	0.726	0.621	0.653	0.687	0.648	0.780	0.658	0.672	0.666	0.728
GLSh5	0.705	0.659	0.616	0.756	0.625	0.654	0.783	0.634	0.701	0.674
GCSh5 + GLSh5	0.786	0.612	0.629	0.775	0.643	0.736	0.747	0.618	0.667	0.701
GCSr5	0.605	0.626	0.625	0.624	0.633	0.646	0.719	0.634	0.659	0.659
GLSr5	0.627	0.648	0.624	0.640	0.630	0.680	0.626	0.629	0.691	0.672
GCSr5 + GLSr5	0.627	0.617	0.615	0.641	0.633	0.668	0.627	0.633	0.641	0.648
GCS	0.729	0.623	0.699	0.691	0.669	0.795	0.732	0.763	0.683	0.797
GLS	0.722	0.623	0.610	0.765	0.624	0.633	0.795	0.643	0.713	0.688
GCS + GLS	0.762	0.631	0.636	0.790	0.674	0.798	0.789	0.678	0.743	0.734
LAESV3D	0.749	0.662	0.633	0.764	0.673	0.690	0.693	0.607	0.747	0.657
LAEDV3D	0.692	0.618	0.612	0.733	0.673	0.640	0.690	0.625	0.710	0.639

**Figure 1:**
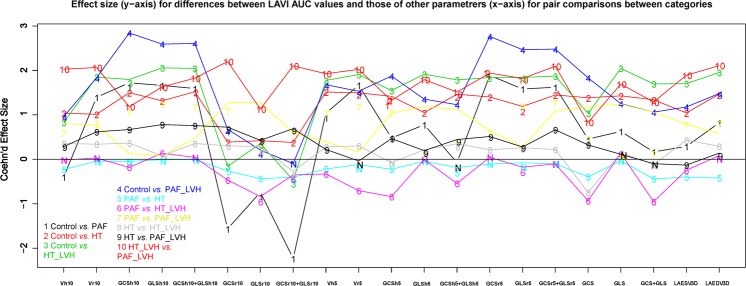
Effect size (*y*-axis) for differences between LAVi’AUC values and those of other parameters (*x*-axis) for pair comparisons between categories. When, for a given pair-wise comparison, LAVi’AUC is better than that of the parameter specified in the abscissa the corresponding effect size is larger than 0, while it is smaller for the opposite situation. “N” symbol indicates comparisons that are not significant.

Some homologous times strain and strain rates alone and in combination, had a better AUC than LAVi and standard 3D strain to detect each group. Abbreviations: LAVi – Left atrial volume index; LAESV3D – Left atrial end systolic volume 3D; LAEDV3D – Left atrial end diastolic volume 3D; Vh10- Volume at tenth homologous time; Vr10- Volume rate at tenth homologous times; Vh5- Volume at fifth homologous time; Vr5- Volume rate at fifth homologous time; GCSh10- Global circumferential strain at tenth homologous times; GLSh10 – Global longitudinal strain at tenth homologous time; GCSh5 – Global circumferential strain at fifth homologous time; GLSh5 – Global longitudinal strain at fifth homologous time; GCSr10 – Global circumferential strain rate at tenth homologous time; GLSr10 – Global longitudinal strain rate at tenth homologous time; GCSr5 – Global circumferential strain rate at fifth homologous time; GLSr5 – Global longitudinal strain rate at fifth homologous time. Figure 1 reports the effect sizes and significance of comparisons of differences between LAVi based AUC and those of the other parameters.

### Evaluation of new 3DSTE parameters at homologous times to detect each group

Receiver operating characteristic (ROC) curves showed a very good performance of all 3DSTE parameters interpolated at homologous times, but only few of them were better of the “shape parameter” LAVi, in differentiating groups in the 10 possible pairwise comparisons. This is shown in Fig. 1 that illustrates the effect size (*y*-axis) for differences between LAVi’AUC values and those of other parametrers (*x*-axis) for pair comparisons between categories. When, for a given pairwise comparison, LAVi’AUC is better than that of the parameter specified in the abscissa, the corresponding effect size is larger than 0, while it is smaller for the opposite situation. Specifically, to distinguish PAF patients in sinus rhythm, with or without LVH (PAF and PAF_LVH) the strain rate variables at pre active time (GCSr10, GLSr10, GCSr10 plus GLSr10) showed a higher AUC value than LAVi (see Table 4). The best classification performance was found for GCSr10 plus GLSr10 for Control *vs* PAF and Control *vs* PAF_LVH (AUC 0.937; 0.925 respectively); notably, strain rate variables better classified each group than standard strain (GCS and GLS alone or combined) except in HT *vs* PAF_LVH and PAF *vs* HT groups (see also Table 4). Concerning Volume and Volume rate at any time only in Control *vs* PAF and in PAF *vs* HT groups they showed a better performance than LAVi, which remained superior in all other pairwise comparisons.

### Reproducibility

Intra-observer variability analysis showed that the reproducibility of our results was always very high (variations smaller than 2%). Traditional global 3DSTE LA parameters showed a very good coefficient of variation: Volume: 1.56%; Circumferential Strain global: 0.79%; Longitudinal Strain global: 0.81%. Inter-observer variability returned also a very good performance being the variation coefficient of traditional global 3DSTE LA parameters always smaller than 5%.

## Discussion

The results obtained in this study show: (i) the presence of impaired LA function in HT and PAF patients with or without LVH, compared with Control subjects; (ii) the ability of functional 3DSTE parameters to detect this LA dysfunction in agreement with morphological enlargement visualized by the most commonly used shape parameter (LAVi); (iii) the high performance of the homologous times’ approach, compared to default 3DSTE parameters (i.e. without interpolation at homologous times) and LAVi, to correctly discriminate between groups in pairwise comparisons, in particular Control *vs* PAF (with or without LVH) (see Table 4).

### Left atrial electromechanical remodeling

LA enlargement and dysfunction are frequently seen in patients with PAF and HT, thus increasing cardiovascular risk and becoming indexes of future cardiovascular events^32,33^. Several studies investigate LA function in terms of shape parameters or 2D strain, but often they evaluated this in a single moment of the entire atrial cycle; moreover, they considered only control subjects versus AF patients^20^. In this setting it is not possible to establish the pathophysiological relation between HT and PAF. For this reason it is very difficult to determine how the presence of impaired LA function was caused by HT. In our study we accurately selected the population (see exclusion criteria) and we used a dynamic parameter that is strain rate to visualize the velocity of deformation. Notably, despite different approaches (triggered by the machine in Schaaf *et al*. and by the homologous time concept in our study), the conclusions were similar. Maybe the best approach would be a multi-modal imaging study to properly analyze the relation between AF, HT and fibrosis, as well as to better classify the different types of patients or predict AF when it is asymptomatic. Impaired LA function depends upon fibroblast’s features as they present a lower elastic capacity with respect to healthy subjects and cooperate to increase atrial stiffness (typical of HT) and to develop an impaired electrical excitability^16^, which represents the pathophysiological base for development of LA dys-synchrony in contraction and relaxation^34^ (typical of PAF).These features are indirectly derived by imaging using the 3DSTE parameters that indicate a lower atrial deformation visualized by either standard strain or strain rates^35–37^. In fact, LA impaired relaxation capability is showed by standard 3DSTE as GCS and GLS, but these latters are unable to give information about LA contraction. On the opposite, the novel 3DSTE strain parameters obtained using the homologous times interpolation approach (GLSh, GCSh, GLSr, GCSr and Vr), allow to detect lower *deformation magnitude* in diastolic phase and lower LA *rates of deformation* during atrial pre-active time (corresponding to early ventricular filling phase).

### Classification ability of standard and novel strain parameters using homologous time interpolation

Diastolic and systolic dysfunction were absent in all groups despite LVH. LAVi measured by 2D echocardiography is currently the main prognostic “shape parameter” to define AF risk in patients with PAF diagnosis^38–41^ or even to suspect silent and asymptomatic episodes. It was possible to identify and characterize alterations of LA deformation in HT and PAF patients, with previous episodes of AF, not only by the exclusive use of classic “shape parameters” (like LAVi) but also by analyzing the “functional parameters” such as 3D strains. Notably, in our study, there was a very good agreement between 2D and 3DSTE parameters: impaired function (detectable by reduction of strains and strain rates) and structural changes (identified by increased LAVi). Both approaches agree for a progressive impaired function from controls to PAF_LVH group (control → HT → HT_LVH → PAF → PAF_LVH). In particular, LAVi, GCS, GLS, and the novel strains GLSh5, GCSh5, GLSh10 and GCSh10 show a progressive increase from Control to PAF subjects with the highest values in PAF_LVH (see Table 3).

Atrial mechanical impairment may not be identifiable by standard echocardiography because 2D variables become clearly pathological only in advanced stages of the remodeling process. 3DSTE, instead, is able to identify and characterize the type of impaired deformation both in patients who had a symptomatic or an asymptomatic episode of PAF, regardless of LVH. To confirm this, we tested different shape and functional parameter to check their capability to classify different groups (see Fig. 1). For each pairwise comparison on groups, effect size of the novel strain’AUC were better (smaller than zero) than LAVi’AUC (zero line) and the other AUC’s parameters (larger than zero). These results show the ability of velocity of deformation to assess LA impaired function. Our results might be explained based on the fact that whereas in HT subjects, the reduction in strain is explained by a lack of homogeneity in relaxation and contraction due to the reduced wall elasticity, in PAF subjects, the decay of deformation parameters depends *also* on changes in electrical properties, with the subsequent development of dys-synchrony in release-contraction. In fact, in HT and PAF subjects the presence of atrial inhomogeneity (lower global strain in different LA segments) plus dys-synchrony (different LA segments reach different global strain values in different times), imply more time to passively return from the maximum atrial volume during telediastolic phase to the minimum one during the end of diastasis, before LA pre-active time. This time corresponds to the event preceding the buster pump phase. These evidences probably suggest that LA strain values are inversely proportional to LA stiffness; therefore the lower strain and strain rates values reached by HT and PAF patients (much more in subject with LVH) are explained by the highest LA stiffness as a consequence of LA remodeling due to underlying common pathophysiological pathways between HT and PAF^16^. Recently, analysis of the LA phasic function as the reservoir and conduction phase, shows that they are independent predictors of AF recurrence after electrical cardioversion^42^. We hypothesize therefore that strains and strain rates could be considered additional “functional” risk factors to be included in a multiparametric risk score to stratify the degree of LA remodeling and consequently PAF development.

### Limitations

This study included patients with significant differences in age, BMI and BSA among different groups; in addition, the total sample size was limited, and the 5 groups did not have the same number of subjects. In order to mitigate this we adopted non parametric tests of significance and tests based on permutations.

Because of this recruitment, groups with ventricular hypertrophy had some differences in 2D diastolic parameters compared with the other groups but within the normal range with an elevated filling pressure without any grade of diastolic dysfunction. Control, HT and HT_LVH patients were in sinus rhythm and without any history of PAF or symptoms. Finally, it is often difficult to determine whether morphology deformation changes are expression of an intrinsic LA disease or whether this is due to a secondary process, i.e. a consequence of pressure overload due to LV impairment. A larger and prospective study is thus warranted with an equal distribution among different groups.

## Materials and Methods

### Study population

The study was conducted after the approval by the “Dipartimento di Scienze Cardiovascolari, Respiratorie, Nefrologiche, Anestesiologiche e Geriatriche, Sapienza–Università di Roma” and submission of the protocol, as an observational spontaneous investigation, to the pertinent local Ethical Committee, called “Institutional Human Ethics Committee, Sapienza University of Rome”. For each patient an informed consent describing the entire methodology and data use was obtained. All methods were carried out in accordance with the ethical guidelines of the Declaration of Helsinki. From October 2013 to May 2016, a total of 130 consecutive individuals were enrolled. Exclusion criteria considered history of ischemic heart disease, LV systolic dysfunction (EF < 50%), moderate-severe valvular disease, presence of pulmonary hypertension, previous cardiac surgery, the absence of sinus rhythm, heart rate higher than 80 b/min or less than 60 b/min, previous interventions for ablating AF, symptomatic or documented AF episodes within the previous 3 months, single episodes of AF, diabetes mellitus. Medical history, 12-lead ECG and 2D echocardiography were performed and interpreted by experienced cardiologists; the study population was divided into 5 groups: 82 subjects with no previous history of AF, without any cardiac symptoms including palpitation, and without any pharmacological therapy, were included in the Control group. Patients with at least one episode of documented AF over 30 s duration or access in the Emergency Department for AF, were divided into two subgroups according to the presence (PAF_LVH, 11 patients) or absence (PAF, 9 patients) of left ventricular hypertrophy (LVH), but both groups had HT. The same subdivision was performed for patients with previous diagnosis of primary HT (at least ten years earlier), and in optimal medical therapy, without LVH (18 patients) and with LVH (10 patients). ECG assessment included presence or absence of P-wave and LVH has been identified by indexed LV mass on 2D transthoracic echocardiography. All patients had the first diagnosis of HT on average about 12 years before and were on therapy for about 8 years on average. Cardiovascular risk factors and pharmacological therapy were collected before undergoing echocardiography; CHA2DS2-VASc score was calculated for each group. (see Table 1).

### Reproducibility

As the same operator (AE) was involved in geometry reconstructions, we performed an intra-observer reproducibility analysis. The left atrium of 7 randomly chosen Controls and 3 (randomly chosen) among the patients’ groups were reconstructed twice at a temporal distance >1 year (long-term). As first, we calculated the coefficient of variation of traditional 3DSTE global strains and LA volumes. Coefficient of variation in percentage (i.e. standard deviation divided by the mean * 100) applied to absolute difference between the two replicas of each individual for the global 3DSTE parameters was used as measure of goodness for reproducibility of classical 3DSTE variables. One of us (GE) digitized the same subjects used for intra-observer reproducibility twice (short-term) at one day of temporal distance between the two replicas. These data were used for a preliminary assessment of short-term inter-observer variability.

### Echocardiography

Standard 2D echocardiography was carried out using Toshiba Artida equipment (Toshiba, Tokyo, Japan) with a PST 30SBT (1–5 MHz) phased array transducer in all patients and Controls. 3D echocardiographic acquisitions were performed immediately following 2D echocardiography, using a commercially available PST-25SX matrix-array transducer (Toshiba medical systems, Tokyo, Japan); LA volume, according to 3DSTE, was reconstructed offline using 3D Wall Motion Tracking software version 2.5 (Toshiba Medical System).

### LA and LV 2D echocardiography

In all groups, cardiac chamber size, LV ejection fraction, LV mass indexed were evaluated according to the European Society of Cardiology guidelines. LV end-sistolic (LVESV) and end-diastolic volume were indexed to body surface area (BSA). Doppler, tissue Doppler (TDI) and heart valves evaluations were performed.

LA volume index (LAVi) was estimated upon apical four- and two- chambers views and afterwards indexed by body surface area (BSA). Pulsed-wave Doppler at the tip of mitral valve was used to infer early (E) and late (A) diastolic filling velocities, E deceleration time, and A duration. Both lateral and septal peak annular velocities in systole (S’) and in early diastole (e’) were measured from the base of the mitral annulus at the insertion of the mitral leaflets, in the septal and lateral points of the 4-chamber view and E/e’ lateral ratio was calculated to evaluate LV filling pressure.

### 3D Speckle tracking

Acquisition was triggered by the electrocardiographic R wave. Within a single breath hold and during a constant RR interval, 6 wedge-shaped sub-volumes were acquired from apical window to create full-volume 3D datasets. The sector widths were as smallest as possible to improve temporal and spatial resolution and to obtain a full-volume LA dataset with optimal border delineation. LA volume upon 3DSTE was measured offline using 3D Wall Motion Tracking software version 2.5 (Toshiba Medical System). 3D echocardiographic data-sets were displayed in the apical 4-chamber and 2-chamber views and 3 short-axis views in basal, mid-atrial and superior LA regions, respectively. In the apical 4-chamber and apical 2-chamber views, the endocardial border was traced by user-controlled setting of multiple reference points, starting from the LA base at mitral valve level and going toward LA apex and excluding LA appendage and pulmonary veins from LA cavity, using conventional 2D grey scale echocardiography.

The epicardial border was adjusted manually or by setting a default thickness for the myocardium. Once LA border was detected at the end-diastolic reference frame, 3D wall-motion tracking, which is based on a 3D block-matching algorithm, was automatically performed by the software.

### Atrial function assessment using “homologous times”

Using 3D wall-motion tracking (3DWMT), 3D strain parameters were recorded, and every parameter was defined according to the direction of the wall deformation**:** longitudinal strain (tangential to endocardial border deformation) and circumferential strain (circumferential deformation). Radial strain (wall thickening or thinning perpendicularly to the endocardial border) was not acquired during atrial cycle because LA thickness wall is too small for reliable measurements. The software divides LA into 16 segments similarly to the LV analysis: 4 apical segments, 6 mid-atrial segments and 6 basal-atrial segments. The reconstruction of the 6 wedge-shaped sub-volumes by 3DSTE software is based on triggering R-wave on ECG, but not always the software correctly detects this wave, and in these cases, the following cycle is not properly centered on R-wave. It follows that the results following software reconstruction are occasionally biased, which determines a biased correlation among 3D parameters and each phase of the atrial cycle.

**Figure 2:**
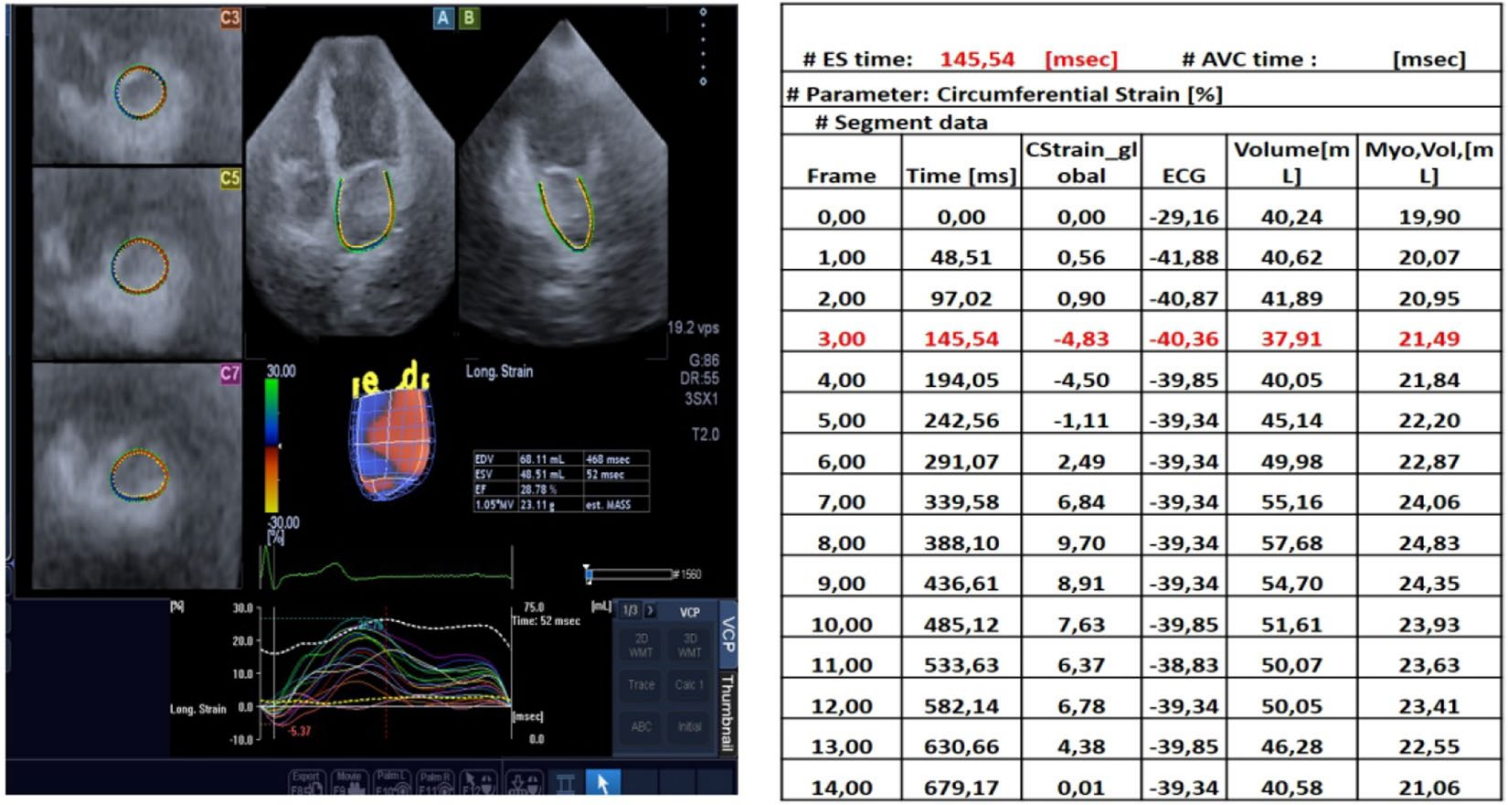
Unequal synchronization between 3D reconstruction and the complete RR interval on surface ECG trace.

Default strains and strain rates parameters available on 3D wall motion software, are not completely appropriate to evaluate correctly each phase of atrial revolution (see Fig. 2). Specifically, default strain and strain rates are dependent on machine frame rate, automatically set at each acquisition (~40 ms on average), and closely correlated to heart rate; each subject is acquired with a different number of frames depending on heart rate determining a low frame acquisition with high heart rate, because the numbers of frames per millisecond are limited by the machine.

**Figure 3:**
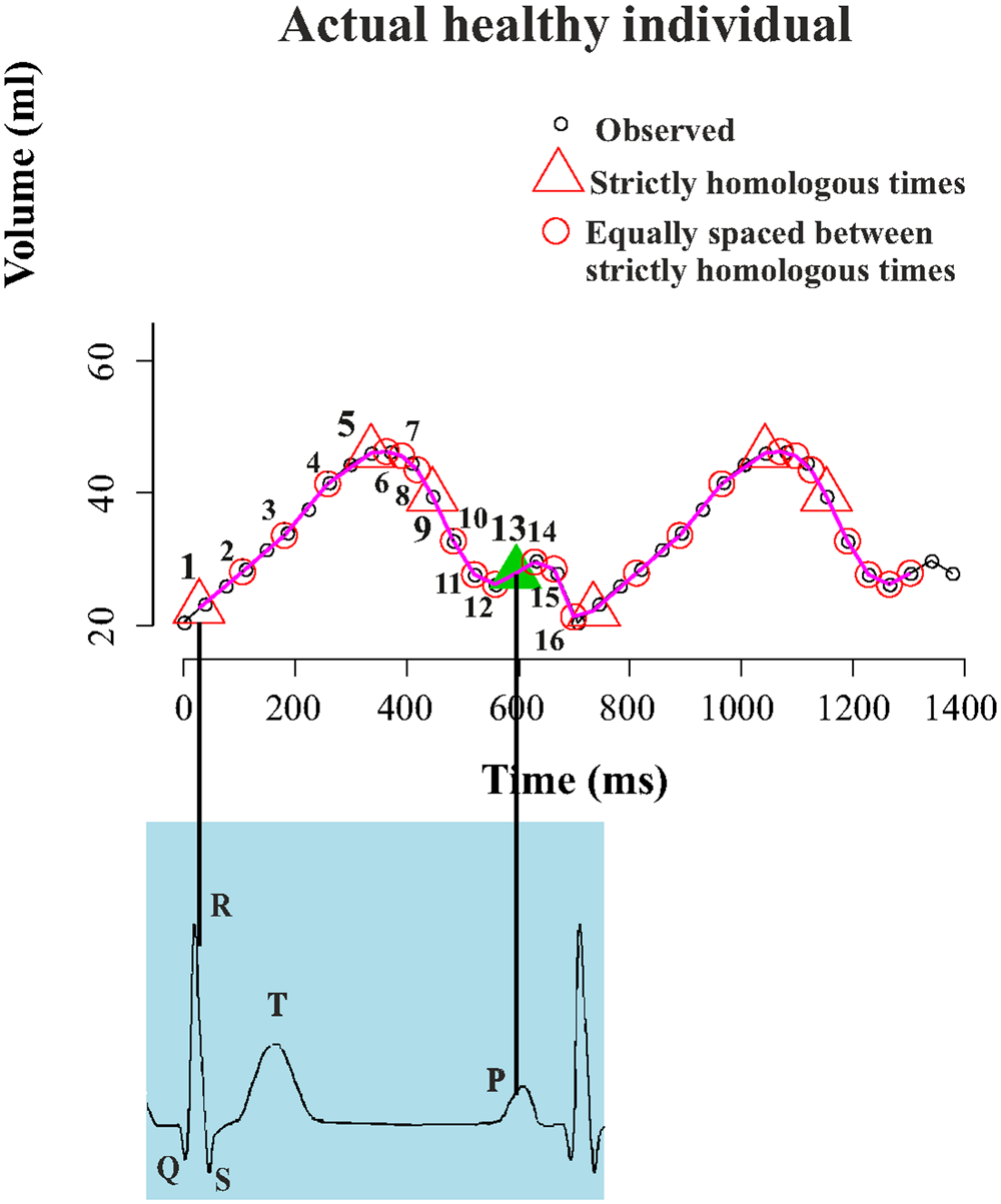
The interpolation at homologous times procedure applied to atrial Volume.

**Figure 4:**
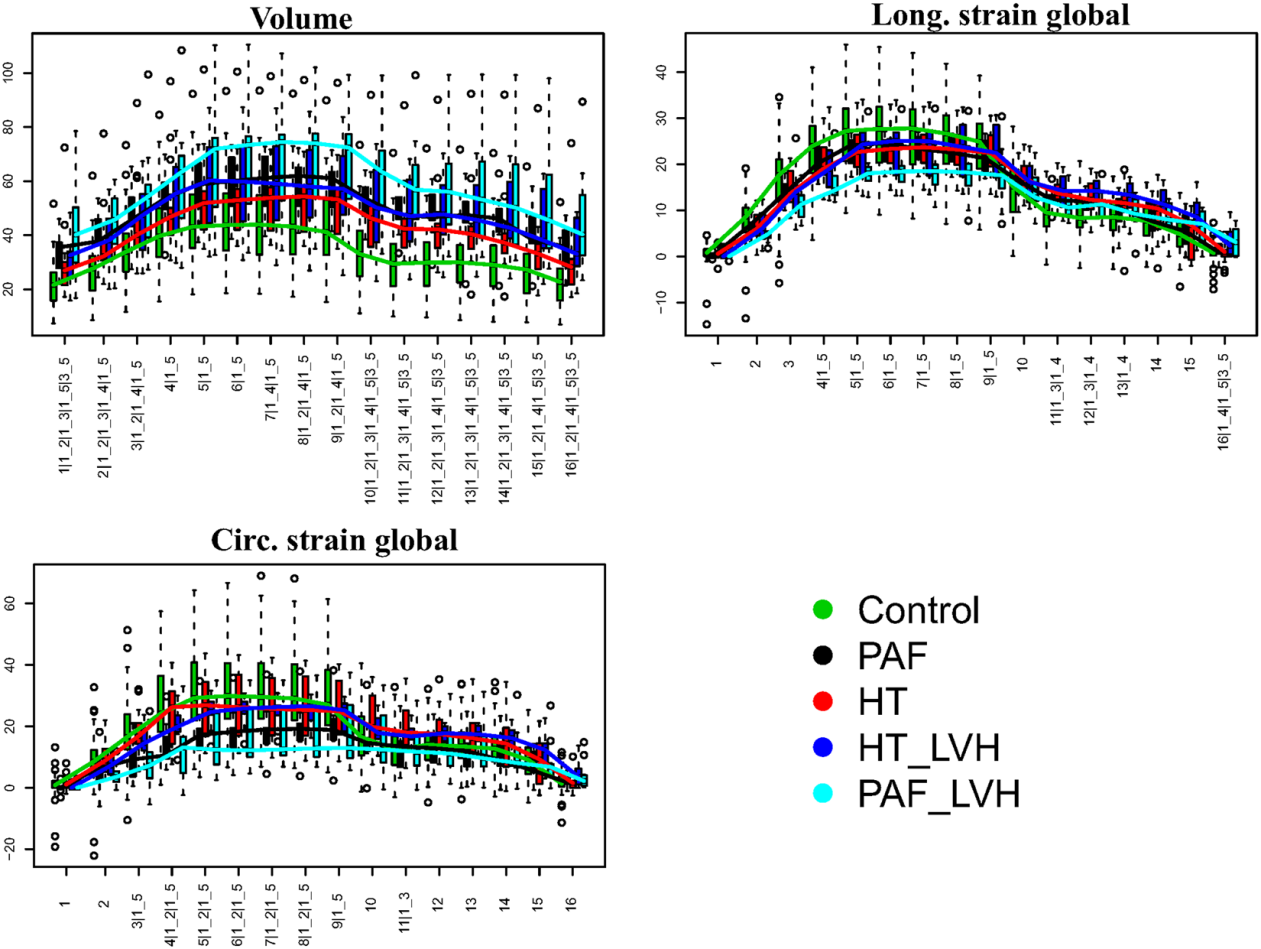
Boxplots showing the distributions of global parameters interpolated at homologous times for the 5 categories considered in this study. Numbers at x-axis thick marks indicate the pair comparisons that are significant under ANOVA in correspondence of each homologous time. Numerical codes: 1: Control; 2: PAF; 3: HT; 4: HT_LVH; 4: PAF_LVH. Abbreviations: PAF: paroxysmal atrial fibrillation; HT: hypertension; HT_LVH: hypertension with left ventricle hypertrophy; PAF_LVH; paroxysmal atrial fibrillation with left ventricle hypertrophy.

To obtain a better assessment of atrial function, we exploited the concept of “homologous times” introduced in^29–31^. We identified 4 strictly homologous times based on some determined physiological events: two mechanical (LV end-systole and mitral valve opening) and two electrical (R wave peak and P wave peak) events. For each individual, we carefully examined 3DSTE clip and we visually recorded the millisecond at which these events occur. One single cycle includes 16 homologous times: the 4 strictly homologous times cited above, and another 12 times obtained by sampling 3 equally spaced times between 2 consecutive strictly homologous ones (see Fig. 3 and 4). For each individual separately we used original time values (as independent variable) and volume and strain parameters outputted by the machine (as dependents) to estimate a cubic spline interpolation function that allows evaluating at the above-mentioned 16 homologous times the dependent variables (volumes and strains). We thus obtained Volume, Global Circumferential strain and Global Longitudinal Strain estimated at homologous times.

This approach allowed an appropriate comparison of any type of 3DSTE variable between different individuals with variable heart rates, because it overcomes the standard strain and strain rate limitation due to heart rate dependency.

### Atrial diastole and pre-active time

**Figure 5:**
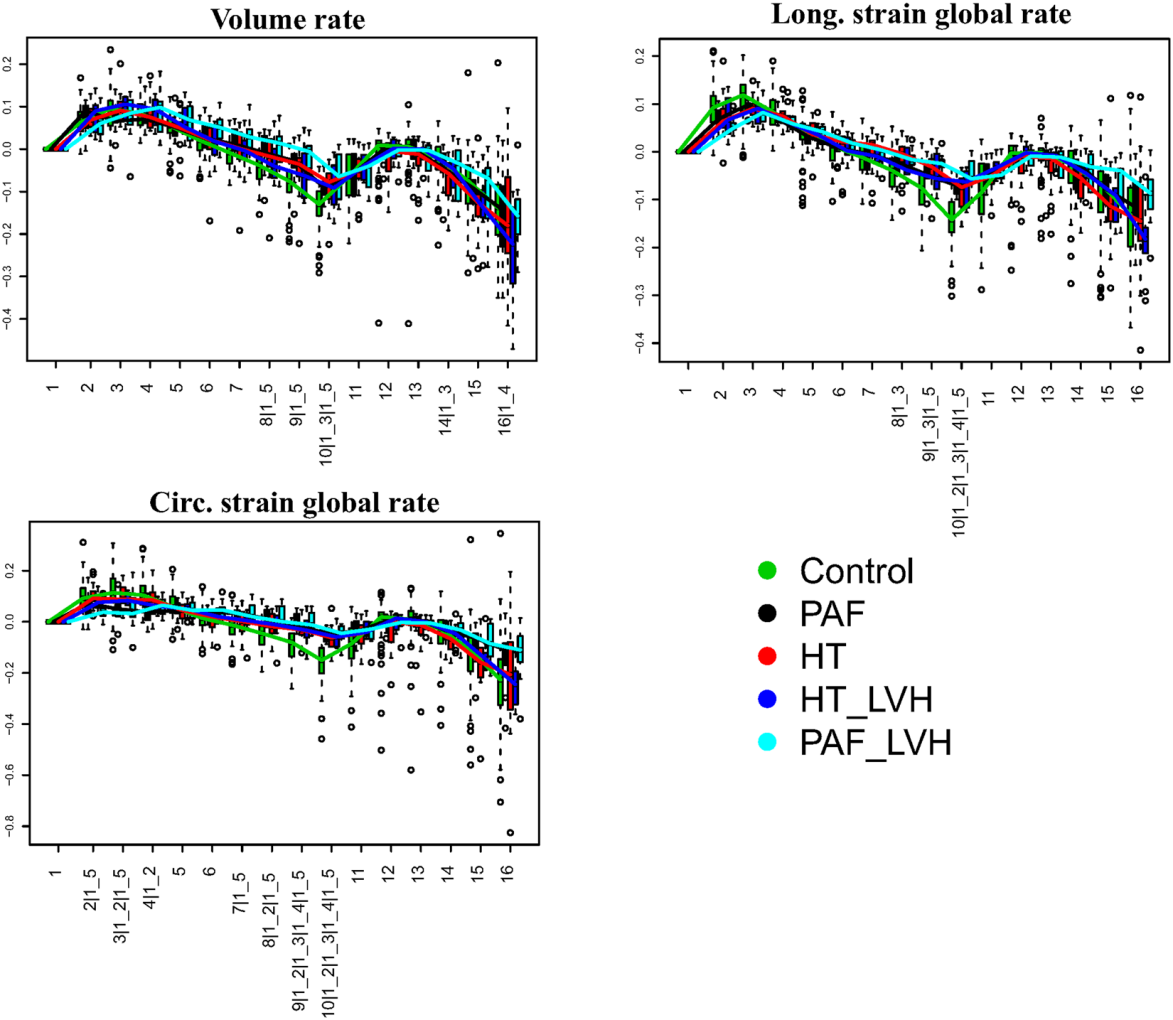
Global parameters’ rates between consecutive homologous times. Numbers at x-axis thick marks indicate the pair comparisons that are significant under ANOVA in correspondence of each homologous time. Numerical codes: 1: Control; 2: PAF; 3: HT; 4: HT_LVH; 4: PAF_LVH. Abbreviations: PAF: paroxysmal atrial fibrillation; HT: hypertension; HT_LVH: hypertension with left ventricle hypertrophy; PAF_LVH; paroxysmal atrial fibrillation with left ventricle hypertrophy.

After interpolation process we considered 2 important times, e.g. fifth (indicated with “h5”) and tenth (indicated with “h10”), which correspond to atrial diastole and pre-active time, respectively. At the former LA reaches maximum dimensions and consequently the maximum deformation just before mitral valve opening. The latter approximately precedes the pre-active phase of atrial diastole, and represents the passive return of the atrium from the maximum volume (LA tele-diastole) to the minimum one, during the early ventricular filling phase, immediately before the atrial active contraction. We calculated global strains, global strain rates, volume and volume rate using volume and strains interpolated at homologous times. The resulting parameters obtained are: (1) Global Longitudinal Strain (GLSh5; GLSh10); (2) Global Circumferential Strain (GCSh5; GCSh10) and (3) Volume (Vh5; Vh10). To derive strain and volume rates we calculated the ratio between parameter’s difference occurring between pairs of consecutive homologous times (5^th^–4^th^ and 10^th^–9^th^) and the corresponding time span. (see Fig. 5). We extracted the following variables: (a) Global Longitudinal strain rate (GLSr5; GLSr10) representing the change per unit time of deformation in the direction tangent to endocardial border; (b) Global Circumferential strain rate (GCSr5; GCSr510) in the direction orthogonal to endocardial border and (c) Volume rate (VSr5; VSr10) representing the volume change per unit time. Strain rates and volume rate are able to provide more functional data about an impaired atrial remodeling than shape parameters or default 3DSTE strains alone. Our procedure mimics the strain rate procedure often performed in 2D albeit with slightly lesser temporal resolution due to the slower 3D acquisition rate.

### Statistical analysis

Continuous variables and results are expressed as mean ± SD. We performed non parametric ANOVA and ROC analysis between all 10 possible pair comparisons among the 5 categories under study. We used Volume, global Circumferential and global Longitudinal strains (interpolated at homologous times) and their rates (derived from interpolated data) for these analyses. We did also the same using the default (i.e. as outputted by the machine) 3D variables i.e. GCS, GLS, GLS + GCS, LAEDV3D, LAESV3D and LAVi.

Due to the unbalanced sample sizes among groups, we opted to perform a pairwise (unpaired) Kruskall-Wallis non parametric test for testing differences among groups. Pairwise deletion techniques have been used to handling casewise missing data. In addition, using Support Vector Machine (SVM) we calculated receiver operating characteristic curve (ROC) to assess classification performance based on all parameters assessed separately: these were: (1)Volume at 10^th^ homologous time, (2)Volume rate at 10^th^ homologous time, (3) Global Circumferential strain at 10^th^ homologous time, (4) Global Longitudinal strain at 10^th^ homologous time, (5) Global Circumferential strain + Global Longitudinal strain at 10^th^ homologous time, (6) Global Circumferential strain rate at 10^th^ homologous time, (7) Global Longitudinal strain rate at 10^th^ homologous time, (8) Global Circumferential strain rate + Global Longitudinal strain rate at 10^th^ homologous time, (9) Volume at 5^th^ homologous time, (10) Volume rate at 5^th^ homologous time, (11) Global Circumferential strain at 5^th^ homologous time, (12) Global Longitudinal strain at 5^th^ homologous time, (13) Global Circumferential strain + Global Longitudinal strain at 5^th^ homologous time, (14) Global Circumferential strain rate at 5^th^ homologous time, (15) Global Longitudinal strain rate at 5^th^ homologous time, (16) Global Circumferential strain rate + Global Longitudinal strain rate at 5^th^ homologous time, (17) Maximum Circumferential strain recorded by the machine, (18) Maximum Longitudinal strain recorded by the machine, (19) Maximum Circumferential strain recorded by the machine + Maximum Longitudinal strain recorded by the machine, (19) Left Atrial End Systolic Volume in 3D, (20) Left Atrial End Diastolic Volume in 3D, (21) Left Atrial Volume Index. We adopted a permutated version of SVM on split data. Control and others groups are unbalanced (i.e. C = 82; PAF = 9; HT = 18; HT_LVH = 10; PAF_LVH = 11 subject); for this reason, we chose to randomly select, as training dataset, the same number of individuals from the designated predictors for each group that are used for learning dictated, in the pair comparison by the category with less subjects. The 10 training sample sizes, for the 10 pairwise comparisons used for both categories involved in the classification exercise are: 6, 15, 7, 8, 6, 6, 6, 7, 8, 7 for Control-PAF, Control-HT, Control-HT_LVH, Control-PAF_LVH, PAF-HT, PAF-HT_LVH, PAF-PAF_LVH, HT-HT_LVH, HT-PAF_LVH, HT_LVH-PAF_LVH respectively.

The remaining test data-sets for each category are then classified ex-novo using the coefficients extracted during the learning procedure. This was repeated 500 times and mean ROC’AUC, sensitivity, specificity and total accuracy were recorded. We also developed a procedure aimed at assessing the significance between ROC results coming from two different parameters: while it is certainly true that some approaches exist to compare ROC curves (e.g. Delong and Delong among others) our ROCs are based on permutations so the randomizations made for different parameters do not include the same sub-samples; in this situation a test aimed at ROC’s comparison would risk to compare curves that are not comparable. We then compared ROCs using all AUC values coming from simulations: we performed an ANOVA on these simulated values using as factor variable a level with 500 cases for one parameter and other 500 for the other one and we reported the corresponding *p*-value and Cohen’d effect size. In this way we can assess which parameter, for a given pairwise category-comparison, yields the best ROC. Level of significance was set at p < 0.05. Given the relatively large sample size (i.e. 500 for each level) in the comparisons of AUC simulated values, one could find a significant *p*-value even for negligible differences; for this reason we urge the reader to evaluate Cohen’d effect size that indicates the strength of AUC differences. Statistical tests were performed by R (software 3.0.2, 2013).

## Conclusions and Perspectives

Several shape parameters have been studied in the literature for LA relative to AF. However, they can show only some specific aspects of atrial remodeling that includes also impaired function that probably begins to take place before manifest anatomical alterations.

Furthermore, in HT and PAF there are a common LA electro-mechanical remodeling also determined by atrial fibrosis^4,10,16^ that have an high functional impact on LA remodeling.

Our findings show that strains and strain rates variables obtained by using the homologous time approach demonstrate anatomical and mechanical LA remodeling in PAF and HT groups, regardless of detectable diastolic dysfunction. Strain rates also suggest a high performance to correctly classify the categories under investigation in comparison with the best 2D “shape parameter” LAVi, and with default 3DSTE variables (GCS, GLS, LAEDV3D and LAESV3D). In particular, a very good performance was found in identifying PAF patients with and without LVH. Finally, there is a potential tendency towards progressive alteration of atrial anatomic-functional relations in HT → PAF → HT_LVH → PAF_LVH.

The identification of LA chamber functional alterations in HT patients without a history of PAF could identify subjects for whom a therapeutic regimen interfering with the wall remodeling could be useful. In fact, delaying or preventing AF onset in these patients may have a huge economic and social impact.

## Supplementary information

Supplementary Table 1

## Data Availability

All datasets are available upon request to the corresponding author (P.E.P.).

## References

[1] Stewart S, Hart CL, Hole DJ, McMurray JJ (2001). Population prevalence, incidence, and predictors of atrial fibrillation in the Renfrew/Paisley study. Heart.

[2] Go AS (2001). Prevalence of diagnosed atrial fibrillation in adults: national implications for rhythm management and stroke prevention: the AnTicoagulation and Risk Factors in Atrial Fibrillation (ATRIA) Study. JAMA.

[3] Miyasaka Y (2006). Secular trends in incidence of atrial fibrillation in Olmsted County, Minnesota, 1980 to 2000, and implications on the projections for future prevalence. Circulation..

[4] Kirchhof P (2016). ESC Guidelines for the management of atrial fibrillation developed in collaboration with EACTS. Eur Heart J..

[5] Bruggenjurgen B, Reinhold T, McBride D, Willich SN (2010). Atrial fibrillation epidemiologic, economic and individual burden of disease. Dtsch Med Wochenschr.

[6] Thrall G, Lane D, Carroll D, Lip GY (2006). Quality of life in patients with atrial fibrillation: a systematic review. Am. J. Med..

[7] Healey JS (2012). Subclinical atrial fibrillation and the risk of stroke. N. Engl. J. Med..

[8] Sanna T (2014). Cryptogenic stroke and underlying atrial fibrillation. N. Engl. J. Med..

[9] January CT (2014). AHA/ACC/HRS guideline for the management of patients with atrial fibrillation: a report of the American College of Cardiology/American Heart Association Task Force on Practice Guidelines and the Heart Rhythm Society. J. Am. Coll. Cardiol..

[10] Yamano Michiyo, Yamano Tetsuhiro, Iwamura Yumi, Nakamura Takeshi, Shiraishi Hirokazu, Shirayama Takeshi, Matoba Satoaki (2017). Impact of Left Ventricular Diastolic Property on Left Atrial Function from Simultaneous Left Atrial and Ventricular Three-Dimensional Echocardiographic Volume Measurement. The American Journal of Cardiology.

[11] Pritchett AM (2005). Diastolic dysfunction and left atrial volume: a population-based study. J. Am. Coll. Cardiol..

[12] Tsang TS (2002). Left ventricular diastolic dysfunction as a predictor of the first diagnosed non valvular atrial fibrillation in 840 elderly men and women. J. Am. Coll. Cardiol..

[13] Allessie M, Ausma J, Schotten U (2002). Electrical, contractile and structural remodeling during atrial fibrillation. Cardiovasc. Res..

[14] Bursteinm B, Nattel S (2008). Atrial fibrosis: mechanisms and clinical relevance in atrial fibrillation. J. Am. Coll. Cardiol..

[15] Frustaci A (1997). Histological substrate of atrial biopsies in patients with lone atrial fibrillation. Circulation..

[16] Verdecchia P, Angeli F, Reboldi G (2018). Hypertension and Atrial Fibrillation: Doubts and Certainties From Basic and Clinical Studies. Circ Res..

[17] Varela M (2017). Novel Computational Analysis of Left Atrial Anatomy Improves Prediction of Atrial Fibrillation Recurrence after Ablation. Front. Physiol..

[18] Bisbal F (2018). Left atrial geometry and outcome of atrial fibrillation ablation: results from the multicentre LAGO-AF study. Eur Heart J. Cardiovasc. Imaging.

[19] Gupta DK (2014). Left atrial structure and function in atrial fibrillation: ENGAGE AF-TIMI 48. Eur. Heart. J..

[20] Schaaf M (2017). Left atrial remodelling assessed by 2D and 3D echocardiography identifies paroxys mal atrial fibrillation. Eur Heart J Cardiovasc Imaging..

[21] Habibi M (2015). Association of left atrial function and left atrial enhancement in patients with atrial fibrillation: cardiac magnetic resonance study. Circ. Cardiovasc. Imaging..

[22] Kuppahally SS (2010). Left atrial strain and strain rate in patients with paroxysmal and persistent atrial fibrillation: relationship to left atrial structural remodeling detected by delayed-enhancement MRI. Circ. Cardiovasc. Imaging.

[23] Mor-Avi V (2012). Real-time 3D echocardiographic quantification of left atrial volume: multicenter study for validation with CMR. JACC Cardiovasc. Imaging..

[24] Perez de Isla L (2014). Quantification of left atrial volumes using three-dimensional wall motion tracking echocardiographic technology: comparison with cardiac magnetic resonance. Eur. Heart J. Cardiovasc. Imaging..

[25] Cameli M, Lisi M, Righini FM, Mondillo S (2012). Novel echocardiographic techniques to assess left atrial size, anatomy and function. Cardiovasc. Ultrasound..

[26] Badano LP (2016). Left atrial volumes and function by three-dimensional Echocardiography: reference values, accuracy, reproducibility, and comparison with twodimensional echocardiographic measurements. Circ. Cardiovasc. Imaging..

[27] Schneider C (2008). Strain rate imaging for functional quantification of the left atrium: atrial deformation predicts the maintenance of sinus rhythm after catheter ablation of atrial fibrillation. Eur. Heart J..

[28] Ravens U (2015). Application of the RIMARC algorithm to a large data set of action potentials and clinical parameters for risk prediction of atrial fibrillation. Med. Biol. Eng. Comput..

[29] Piras P (2014). 4D-analysis of left ventricular cycle using Procrustes motion analysis. Plos One..

[30] Piras P (2016). Left atrial trajectory impairment in Hypertrophic Cardiomyopathy disclosed by Geometric Morphometrics and Parallel Transport. Sci Rep..

[31] Madeo A (2015). A new 4D trajectory-based approach unveils abnormal LV Revolution Dynamics in Hypertrophic Cardiomyopathy. PLoSONE.

[32] Hoit BD (2014). Left atrial size and function: role in prognosis. J. Am. Coll. Cardiol..

[33] Onishi N (2014). Comparison between left atrial features in well-controlled Hypertensive patients and normal subjects assessed by three dimensional speckle tracking echocardiography. J. Cardiol..

[34] Mochizuki A (2013). Assessment of left atrial deformation and synchrony by three- dimensional speckle-tracking echocardiography: comparative studies in healthy subjects and patients with atrial fibrillation. J. Am. Soc. Echocardiogr..

[35] Chadaide S (2013). Three-Dimensional Speckle tracking Echocardiography-Derived Left atrial Strain Parameters Are reduced in Patients with Atrial Fibrillation. Echocardiography..

[36] Inaba Y (2005). Strain rate imaging for noninvasive functional quantification of the left atrium: comparative studies in controls and patients with atrial fibrillation. J. Am. Soc. Echocardiogr..

[37] Toh N (2010). Left atrial volume combined with atrial pump function identifies hypertensive patients with a history of paroxysmal atrial fibrillation. Hypertension..

[38] Tsang TS (2001). Left atrial volume: important risk marker of incident atrial fibrillation in 1655 older men and women. Mayo Clinic Proc..

[39] Wu VC (2013). Prognostic value of LA Volumes assessed by transthoracic 3D echocardiography: comparison with 2D echocardiography. JACC Cardiovasc. Imaging..

[40] Nappo R (2015). Quantitative assessment of atrial conduit function: a new index of diastolic dysfunction. Clin. Res. Cardiol..

[41] Casaclang-Verzosa G, Gersh BJ, Tsang TSM (2008). Structural and functional remodeling of the left atrium. Clinical and therapeutic implications for atrial fibrillation. J. Am. Coll. Cardiol..

[42] Giubertoni A (2019). Atrial conduit function quantitation precardioversion predicts early arrhythmia recurrence in persistent atrial fibrillation patients. J. Cardiovasc. Med..

