# Supplementary material for: Improving performance of 3D speckle tracking in arterial hypertension and paroxysmal atrial fibrillation by using novel strain parameters
Source: Sci Rep. 2019 May 14;9:7382. doi: 10.1038/s41598-019-43855-7 (PMC6517438; doi:10.1038/s41598-019-43855-7)
Supplement: Supplementary file 1 — Supplementary Table 1 [file 41598_2019_43855_MOESM1_ESM.pdf]

Improving performance values of 3D speckle tracking in arterial hypertension and paroxysmal atrial fibrillation by using novel strain parameters

G.Esposito[1], P.Piras[1,2], A.Evangelista[3],V.Nuzzi[1], P.Nardinocchi[2], G.Pannarale[1],

C.Torromeo[1] and P.E.Puddu[1*]

[1] Dipartimento di Scienze Cardiovascolari, Respiratorie, Nefrologiche, Anestesiologiche e Geriatriche, Sapienza –Università di Roma, Rome, Italy. [2] Dipartimento di Ingegneria Strutturale e Geotecnica, Sapienza – Università di Roma, Rome, Italy. [3] Ospedale San Giovanni Calibita Fatebenefratelli – Isola Tiberina, Rome, Italy. *Correspondence and requests for materials should be addressed to P.E.P. (email: paoloemilio.puddu@uniroma1.it)

Supplementary Table 1. Kruskal Wallis non parametric test with Benjamini-Hockberg p-value correction of left atrial volume index, standard 3D strain and 3D strain rate variables calculated by homologous times approach at pre active time and atrial diastole of our population divided by groups. LAVi – Left atrial volume index; LVMASSi – left ventricular mass index; GCS – Global circumferential strain; GLS – Global longitudinal strain; LAESV3D – Left atrial end systolic volume 3D; LAEDV3D – Left atrial end diastolic volume 3D; Vh10- Volume at tenth homologous time; Vr10- Volume rate at tenth homologous times; Vh5- Volume at fifth homologous time; Vr5- Volume rate at fifth homologous time; GCSh10- Global circumferential strain at tenth homologous times; GLSh10 – Global longitudinal strain at tenth homologous time; GCSh5 – Global circumferential strain at fifth homologous time; GLSh5 – Global longitudinal strain at fifth homologous time; GCSr10 – Global circumferential strain rate at tenth homologous time; GLSr10 – Global longitudinal strain rate at tenth homologous time; GCSr5 – Global circumferential strain rate at fifth homologous time; GLSr5 – Global longitudinal strain rate at fifth homologous time; * Significative P-value set at <0.05.

**P - Value**			**2D ECHOCARDIOGRAPHIC VARIABLES**		
	CONTROL	PAF	HT	HT_LVH	PAF_LVH
LAVi					
CONTROL	1	**0.008***	**0.020***	**0.009***	**<0.001***
PAF	**0.008***	1	1	1	1
HT	**0.020***	1	1	1	0.223
HT_LVH	**0.009***	1	1	1	1
PAF_LVH	**<0.001***	1	0.223	1	1
LVMASSi					
CONTROL	1	0.777	1	**<0.001***	**<0.001***
PAF	0.777	1	1	**0.002***	**0.03***
HT	1	1	1	**<0.003***	**<0.001***
HT_LVH	**<0.001***	**0.002***	**<0.003***	1	1
PAF_LVH	**<0.001***	**0.03***	**<0.001***	1	1

**P - Value**	**STANDARD STRAIN VARIABLES AND 3D VOLUME**				
	CONTROL	PAF	HT	HT_LVH	PAF_LVH
GLS					
CONTROL	1	1	0.985	1	**0.004***
PAF	1	1	1	1	1
HT	0.985	1	1	1	0.644
HT_LVH	1	1	1	1	0.888
PAF_LVH	**0.004***	1	0.644	0.888	1
GCS					
CONTROL	1	**0.022***	1	1	**0.018***
PAF	**0.022***	1	0.388	1	1
HT	1	0.388	1	1	0.438
HT_LVH	1	1	1	1	1
PAF_LVH	**0.018***	1	0.438	1	1
LAEDV3D					
CONTROL	1	0.064	0.304	0.595	**0.006***
PAF	0.064	1	1	1	1
HT	0.304	1	1	1	1
HT_LVH	0.595	1	1	1	1
PAF_LVH	**0.006***	1	1	1	1
LAESV3D					
CONTROL	1	**0.003***	0.146	0.171	**<0.001***
PAF	**0.003***	1	1	1	1
HT	0.146	1	1	1	1
HT_LVH	0.171	1	1	1	
PAF_LVH	**<0.001***	1	0.773	1	1

**P - Value**	**3D STRAIN VARIABLES AND VOLUME AT HOMOLOGOUS TIMES**				
TENTH HOMOLOGOUS TIME (Pre active time)					
	CONTROL	PAF	HT	HT_LVH	PAF_LVH
GCSh10					
CONTROL	1	0.600	0.410	0.600	0.600
PAF	0.600	1	0.410	0.410	0.770
HT	0.410	0.410	1	0.600	0.410
HT_LVH	0.600	0.410	0.600	1	0.600
PAF_LVH	0.600	0.770	0.410	0.600	1
GLSh10					
CONTROL	1	0.690	0.560	0.690	0.840
PAF	0.690	1	0.690	0.840	0.690
HT	0.560	0.690	1	0.690	0.560
HT_LVH	0.690	0.840	0.690	1	0.690
PAF_LVH	0.840	0.690	0.560	0.690	1
Vh10					
CONTROL	1	**<0.001***	**<0.001***	**0.002***	**<0.001***
PAF	**<0.001***	1	0.140	0.780	0.280
HT	**<0.001***	0.140	1	0.340	0.04*
HT_LVH	**0.002***	0.780	0.340	1	0.340
PAF_LVH	**<0.001***	0.280	0.04*	0.340	1

FIFTH HOMOLOGOUS TIME (Atrial diastole)

	CONTROL	PAF	HT	HT_LVH	PAF_LVH
GCSh5					
CONTROL	1	**<0.001***	0.251	0.173	**<0.001***
PAF	**<0.001***	1	0.134	0.069	0.88
HT	0.251	0.134	1	0.548	0.069
HT_LVH	0.173	0.069	0.548	1	0.164
PAF_LVH	**<0.001***	0.880	0.069	0.164	1
GLSh5					
CONTROL	1	0.202	0.187	0.421	**0.039***
PAF	0.202	1	0.955	0.968	0.084
HT	0.187	0.955	1	0.949	0.082
HT_LVH	0.421	0.968	0.949	1	0.197
PAF_LVH	**0.039***	0.084	0.082	0.197	1
Vh5					
CONTROL	1	**0.031***	0.120	**0.041***	**0.031***
PAF	**0.031***	1	0.379	0.968	0.463
HT	0.120	0.379	1	0.379	0.347
HT_LVH	**0.041***	0.968	0.379	1	0.568
PAF_LVH	**0.031***	0.463	0.347	0.568	1

**P - Value**	**3D STRAIN RATE VARIABLES AND VOLUME RATE AT HOMOLOGOUS TIMES**				
TENTH HOMOLOGOUS TIME (Pre active time)					
	CONTROL	PAF	HT	HT_LVH	PAF_LVH
GCSr10					
CONTROL	1	**0.001***	**0.001***	**0.001***	**0.001***
PAF	**0.001***	1	0.320	0.670	0.670
HT	**0.001***	0.320	1	0.760	0.290
HT_LVH	**0.001***	0.670	0.760	1	0.500
PAF_LVH	**0.001***	0.670	0.290	0.500	1
GLSr10					
CONTROL	1	**0.001***	**0.001***	**0.001***	**0.001***
PAF	**0.001***	1	0.780	0.680	0.051
HT	**0.001***	0.780	1	0.680	0.051
HT_LVH	**0.001***	0.680	0.680	1	0.247
PAF_LVH	**0.001***	0.051	0.051	0.247	1
Vr10					
CONTROL	1	**0.040***	**0.008***	0.103	**0.008***
PAF	**0.040***	1	0.955	0.780	0.588
HT	**0.008***	0.955	1	1	0.955
HT_LVH	0.103	0.955	1	1	0.588
PAF_LVH	**0.008***	0.588	0.588	0.588	1

FIFTH HOMOLOGOUS TIME (Atrial diastole)

	CONTROL	PAF	HT	HT_LVH	PAF_LVH
GCSr5					
CONTROL	1	0.930	0.720	0.930	0.930
PAF	0.930	1	1	1	1
HT	0.720	1	1	0.930	1
HT_LVH	0.930	1	0.930	1	1
PAF_LVH	0.930	1	1	1	1
GLSr5					
CONTROL	1	0.880	0.880	0.880	0.880
PAF	0.880	1	0.880	0.880	0.880
HT	0.880	0.880	1	0.880	0.880
HT_LVH	0.880	0.880	0.880	1	0.970
PAF_LVH	0.880	0.880	0.880	0.970	1
Vr5					
CONTROL	1	1	1	1	1
PAF	1	1	1	1	1
HT	1	1	1	1	1
HT_LVH	1	1	1	1	1
PAF_LVH	1	1	1	1	1
